# Longus colli tendinitis. A review of literature and case series

**DOI:** 10.1051/sicotj/2017032

**Published:** 2017-06-28

**Authors:** Ahmed Shawky, Belal Elnady, Essam El-Morshidy, Wael Gad, Ali Ezzati

**Affiliations:** 1 Department of Orthopedics and Trauma Surgery, Assiut University Hospitals 71111 Assiut Egypt; 2 Spine Center, Helios Klinikum Erfurt Nordhaeuser street 74 99089 Erfurt Germany

**Keywords:** Longus colli tendinitis, Retropharyngeal tendinitis, Prevertebral tendinitis, Calcium hydroxyapatite deposition disease, Neck pain

## Abstract

*Purpose*: To increase the awareness of longus colli tendinitis (LCT) among spine specialists and to present a practical overview of diagnostic and treatment options, so that unnecessary interventions are avoided. Five sample cases from a German spine center will also be presented.

*Methods*: Literature review and case series. A PubMed search was performed in May 2015, and the articles found were reviewed for clinical presentation, investigations, and treatment. The frequency of publication of LCT cases and the specialty of journals were also noted. Recent cases treated in our institution were also reviewed. The clinical findings, investigations, and therapeutic interventions were summarized.

*Results*: The PubMed search from May 2015 found 104 articles, published over 51 years, on the topic of LCT. Only four were published in spine journals. A review of this literature yielded a total of 242 cases. The classic clinical triad included neck pain, limitation of movements, and swallowing complaints. C-reactive Protein (CRP) values were available in 21 cases (mean 23.66 mg/dL). A contrast-enhanced computed tomography (CT) scan was the best diagnostic modality. LCT is usually a self-limiting condition, but non-steroidal anti-inflammatory drugs (NSAIDs) may help alleviate discomfort. Five cases of LCT were diagnosed and treated in our center over the past three years.

*Conclusions*: LCT, which is uncommon and has non-specific symptoms, is often referred to spine centers. Spine specialists should be aware of its clinical presentation and radiographic findings in order to avoid unnecessary interventions. The condition is self-limiting and can be treated conservatively.

## Introduction

Calcific aseptic tendinitis of the longus colli, also known as calcific retropharyngeal tendinitis or calcific prevertebral tendinitis, was first described in Swedish by Fahlgren and Loefstedt in 1963 [1] and then in English by Hartley in 1964 [2]. The pathophysiological basis of the condition is a foreign-body inflammatory response to deposited calcium hydroxyapatite in the superior oblique tendon of the longus colli [3]. Although the first description of the condition in English was published in the Journal of Bone and Joint Surgery (Am), later there were few articles published in such specialized orthopedic and spine journals. This rare and often misdiagnosed condition is thus relatively unknown among spine specialists.

Longus colli tendinitis (LCT) is not listed among the differential diagnoses for neck pain in many textbooks, which generally only include more common possibilities such as inflammatory, infectious, and rheumatic conditions [4]. Furthermore, it is primarily seen in emergency departments and in the primary health care sector by family physicians, chiropractors, physiotherapists, speech language pathologists and, occasionally, by otolaryngologists. As patients commonly present with acute or subacute neck pain, rigidity and limitation of cervical movements, they are frequently referred to orthopedic or spine departments. Spine surgeons (orthopedic and neurosurgeons) should be aware of LCT as a possible differential diagnosis in order to avoid unnecessary interventions after investigation and exclusion of more serious conditions.

The aim of this review is to increase the awareness of LCT among spine surgeons. We present here five cases of LCT from our institution, and we also review the literature and summarize information regarding clinical findings and useful diagnostic tools. To the best of our knowledge, this is the first comprehensive review of LCT papers and cases published between its first recognition as a condition in 1964 up until 2015 and is the first summary that puts the information contained in these papers into a practical context.

## Materials and methods

### Literature review

A PubMed search was performed in May 2015 using “longus colli tendinitis”, “prevertebral tendinitis”, and “retropharyngeal tendinitis” as keywords and covered the years from 1964 until 2015. Only English-language, full-text articles were reviewed. The following data were extracted and recorded: number of cases, epidemiological characteristics, symptoms, examination findings, diagnostic investigations (laboratory and imaging), therapeutic measures, and duration until resolution of symptoms. The collected data were summarized in order to help develop possible diagnostic and therapeutic guidelines for LCT. Specialty and subspecialty journals were also reviewed to determine the number of LCT cases per publication, per year from 1964 to 2015.

### Clinical cases

Five cases of LCT have been diagnosed in our institution over the last three years. Here we describe the presentation, therapeutic interventions, and clinical course. Examples of key imaging studies are also provided. None of the below-mentioned cases reported any history of recent trauma or whiplash injury.

Consent was obtained from the patients for the reporting of their cases.

### Case 1

**Figure 1.**
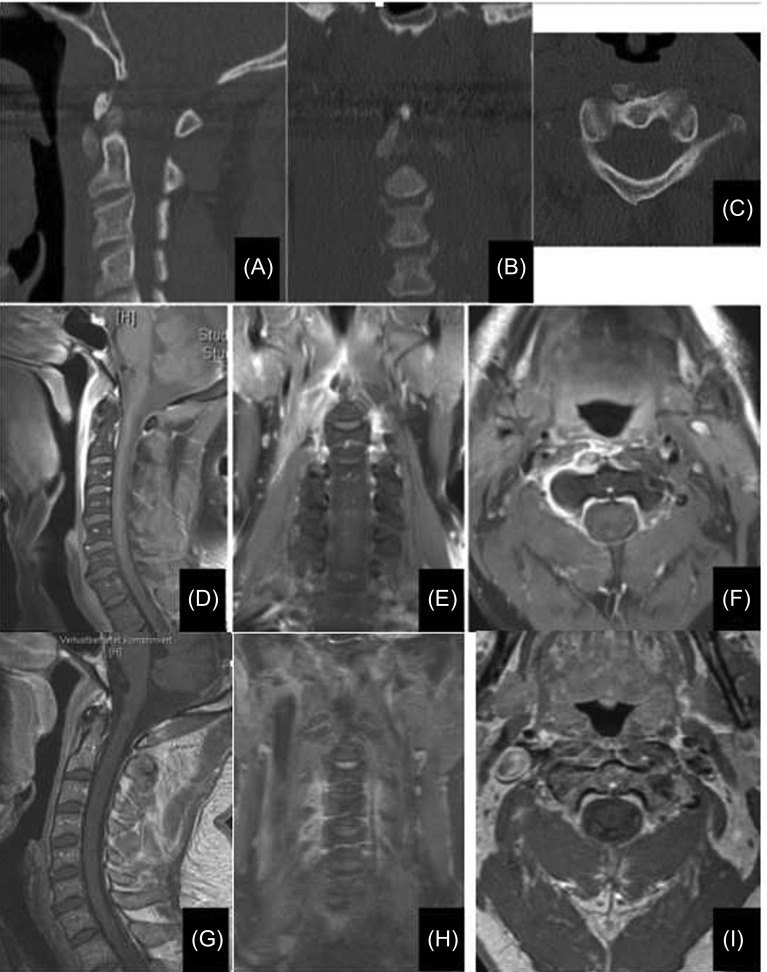
(A–C) CT scans showing calcifications at longus colli origin anterior to C1/2, measuring about 14 mm in the superior-inferior diameter and about 7 mm in the anteroposterior diameter. (D–F) Prevertebral swelling and minimal fluid collection extending from skull base caudally to C4, maximally at the middle of C2 (up to 7 mm). Musculus longus colli on the right side is edematous especially at its insertion at C1 and C2 vertebrae. No other sites of inflammation in the ENT area. The findings correspond to right-sided longus colli tendinitis. (G–I) A follow-up MRI with the same sequences shows the obvious regression of the soft tissue swelling as well as the contrast enhancement. *Note*: the MRIs were done using two different devices and the mostly corresponding cuts were selected for the comparison between that at presentation and at follow-up.

A 45-year-old lady presented to the emergency room (ER) with severe neck pain radiating to the left shoulder and arm, torticollis, and dysphagia for about two days. A clinical examination revealed limited cervical movement in all directions with exacerbation of pain on extension of the head. The primary clinical diagnosis in ER was cervicobrachialgia. The laboratory investigations revealed a leukocyte count of 11.5 × 10^3^/mm^3^ (normal range 3.80–9.80 × 10^3^/mm^3^) and C-reactive Protein (CRP) value of 7.8 mg/dL (normal range 0–5.0 mg/dL). Computed tomography (CT) and magnetic resonance imaging (MRI) were done and showed prevertebral soft tissue swelling and calcifications anterior to C1 at the insertion of the longus colli especially affecting the left side (Figure 1). After radiological confirmation of the diagnosis, non-steroidal anti-inflammatory drugs (NSAIDs) and a soft cervical collar were prescribed. The patient showed complete remission of symptoms after five days.

### Case 2

A 50-year-old man was referred to our hospital with severe occipital headache (VAS 9), odynophagia. The CRP was 38 mg/dL. The main differential diagnosis was a retropharyngeal abscess, so the patient was first admitted to the ENT department where a clinical examination revealed posterior pharyngeal edema. Antibiotics and anti-inflammatory drugs were prescribed. A contrast-enhanced CT was done and revealed the prevertebral calcifications. The neuroradiologist suggested the diagnosis of longus colli tendinitis that was confirmed with contrast-MRI. A soft cervical collar and analgesics were prescribed. Symptoms rapidly improved after three days that the patient could be discharged. The patient reported complete remission of symptoms after seven days.

### Case 3

A 63-year-old lady was presented to the ER with acute neck pain and dysphagia. She was afebrile. Laboratory investigations showed elevated CRP (39.7 mg/dL). MRI with contrast was done and showed inflammatory changes and contrast enhancement at the insertion of longus colli without prevertebral effusion. CT revealed the calcifications and confirmed the diagnosis of LCT.

### Case 4

A 48-year-old man referred to our center with severe left-sided neck pain and dysphagia. Severe limitation of movements in all directions was evident on examination. The leukocyte count and CRP were elevated (15.6 × 10^3^/mm^3^ and 51.3 mg/dL consecutively). Contrast-MRI revealed prevertebral edema, prevertebral effusion, and bone marrow edema of the anterior arch of atlas at the insertion of the upper oblique fibers of the left longus colli muscle mimicking spondylitis. CT scan was done and revealed the prevertebral calcifications. Under NSAIDs the inflammatory parameters were decreasing. Soft cervical collar helped to alleviate discomfort. Remission of symptoms was complete after 10 days.

### Case 5

**Figure 2.**
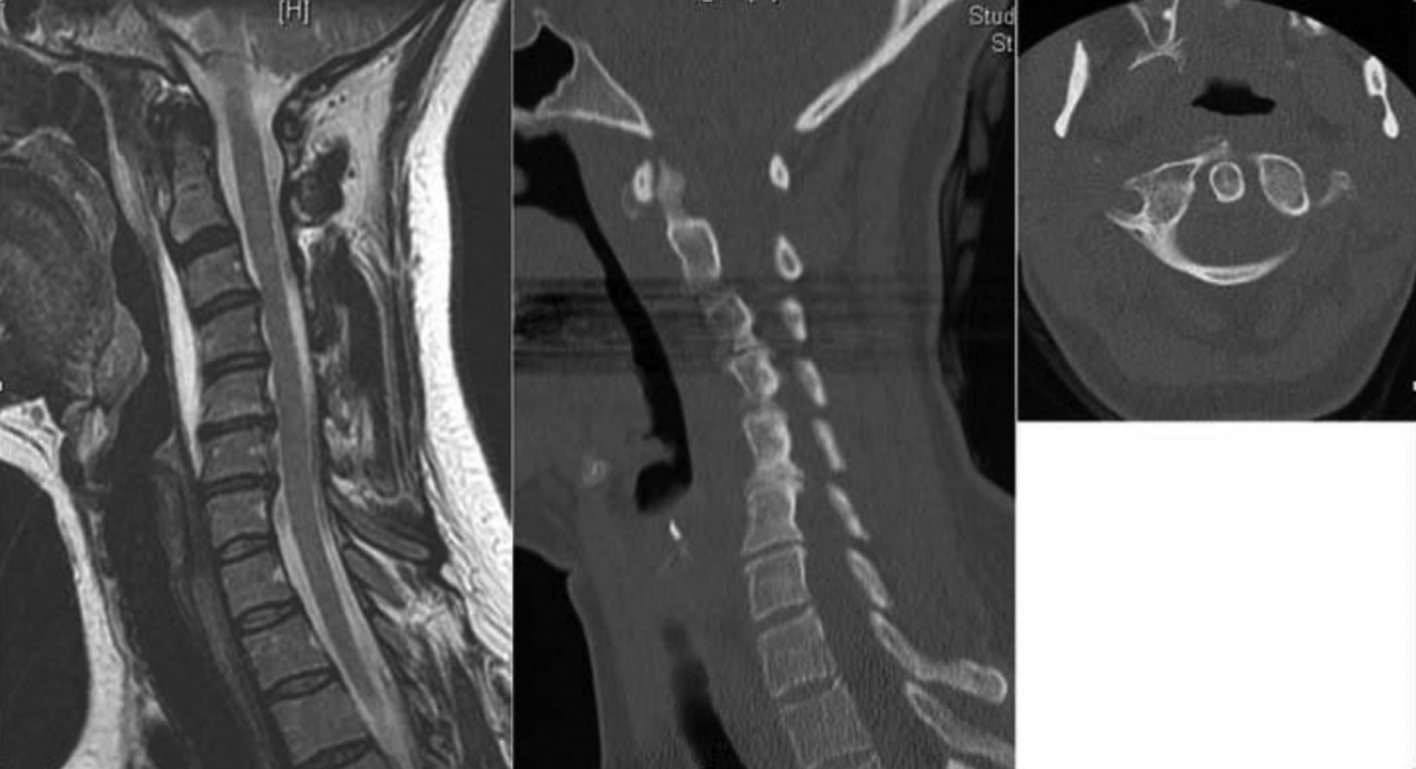
MRI and CT scans of another case showing loss of cervical lordosis, even kyphotic cervical spine, and prevertebral fluid collection extending from skull base caudally to C5, maximally anterior to C4. The CT scan shows the faint small calcification in front of C1, mainly on the right side.

A 51-year-old lady presented to the ER with neck pain and rigidity. She had normal body temperature and normal leukocyte count and CRP. CT and MRT revealed calcifications at C1/2 level, prevertebral effusion, and soft tissue edema extending to the level of C4 (Figure 2). The case was diagnosed as LCT and NSAIDs were prescribed. Symptoms completely improved after two weeks.

Table 1 summarizes the epidemiological, clinical, laboratory, and diagnostic findings in the five cases from our institution.

**Table 1.** Epidemiological, clinical, and investigatory findings of our case series.

	Case 1	Case 2	Case 3	Case 4	Case 5
Age (years)	45	50	63	48	51
Sex	Woman	Man	Woman	Man	Woman
Neck pain (VAS)	+ (8)	+ (9)	+ (7)	+ (8)	+ (8)
Limited ROM*	+	+	+	+	+
Swallowing complaints	+	+	+	+	+
Other complaints	Torticollis, radiating pain in left arm	Occipital headache		Left-sided neck pain	
Primary diagnosis in ER	Cervical disc prolapse	Retropharyngeal abscess	Cervical pain syndrome	Meningitis/Spondylitis	Cervical pain syndrome
Body temperature (°C)	37.2	37	37.2	37	37.1
Leukocyte count** (×10^3^/mm^3^)	11.5	9.77	9.07	15.6	7.6
CRP***(mg/dL)	7.8	38	39.7	51.3	3.3
Calcifications	Yes	Yes	Yes	Yes	Yes
Soft tissue swelling	Yes	Yes	Yes	Yes	Yes
Effusion	Yes	Yes	No	Yes	Yes
Duration till subsidence (days)	5	7	12	10	14

VAS = Visual Analogue Scale for neck pain at presentation,

* ROM = range of motion,

** Leukocyte count (normal range 3.80–9.80 × 10^3^/mm^3^),

*** CRP = C-reactive protein (normal range 0–5 mg/dL).

## Results

### Literature review

**Figure 3.**
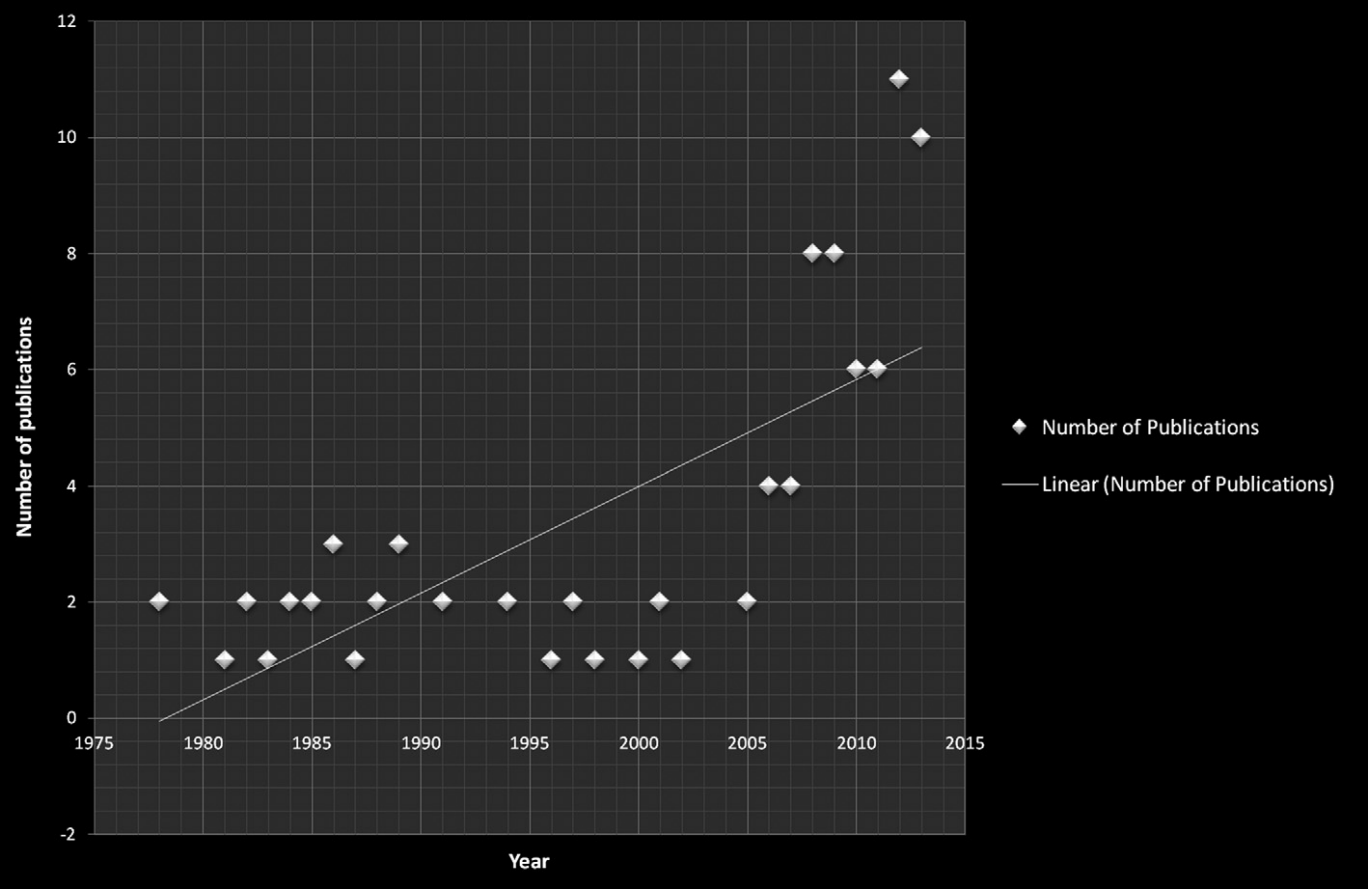
The progressive increase of number of publications per year over the last 40 years.

Review of the literature revealed a total of 104 papers published between 1964 and May 2015. Twenty-three non-English articles were excluded, thus, 81 articles were available for review. The majority of these articles (64 articles) were published between 2008 and 2015 (Figure 3). Twenty-eight articles were published in radiology and neuroradiology journals, 20 were published in otolaryngology journals, and 12 in neurology journals (Table 2). Of note, only four papers were published in spine-specific journals and five in orthopedic journals. In the reviewed literature, a total of 242 cases (129 females and 113 males) were reported with a mean age of 43 years (range 21–81 years), although Benanti et al. reported on a five-year-old boy with suspected LCT [5]. Neck pain was a presenting symptom in 100% of cases. The limitation of cervical movement was evident in 98.3% of cases. Swallowing difficulties such as dysphagia and/or odynophagia, sometimes just a globus sensation of a lump in throat, were present in 83.72% of cases (in 36 cases (15.38%) no data was available regarding swallowing complaints). The body temperature was available in 36 cases with a mean of 37.54 °C (range 36.2–38.9 °C). In 39 cases, descriptive terms such as “normal body temperature” or “no fever” were used.

**Table 2.** Distribution of publications to the different specialties and subspecialties.

Specialty	Number of articles
Radiology and neuroradiology	28
Otolaryngology	20
Neurology	12
Emergency medicine	10
Rheumatology	8
Orthopedics	5
Spine	4
Others	17
Total	104

Some laboratory information was generally provided in the cases reported. The leukocyte count was available in 48 cases with a mean of 11.58 × 10^3^/mm^3^ (range 5.3–21 × 10^3^/mm^3^), the term “normal” was used to describe the leukocyte count in 10 cases, while in four cases the leukocyte counts were described as “increased” but no exact values were provided. Erythrocyte sedimentation rate (ESR) values were available in 45 cases with a mean of 31.57 mm/h (range 5–98 mm/h). CRP values were available in 21 cases with a mean of 23.66 mg/dL (range 0.3–91.3 mg/dL). In 15 cases the CRP values were normal.

Computer tomography (CT) and Magnetic resonance imaging (MRI) were the diagnostic imaging modalities described in the later literature. CT was used for diagnosis in 88 cases, and MRI was used in 51 cases. Before 1986, the diagnosis was mainly based on history, clinical data, plain X-rays and, in few cases, polytomography. In those cases which used CT or MRI, calcifications were evident in 185 cases (about 76.44%), and in 190 (78.51%) cases, prevertebral soft tissue swelling or effusion was present. In 14 (5.7%) cases, there was no comment on the presence of effusion or soft tissue edema, while in six cases soft tissue swelling or effusion was noted to be absent. In cases where CT or MRI was not done, an increased prevertebral soft tissue shadow on the lateral plain X-ray of the cervical spine was taken to indicate soft tissue swelling or effusion.

There were reports of unnecessary interventions or treatments. In five cases, fine needle aspiration from the retropharyngeal space was done. In another five cases, unnecessary surgical intervention was performed due to misdiagnosis [6, 7]. Antibiotics were given in 32 cases (about 14%). With the increased use of CT or MRI, the occurrence of unnecessary interventions or inappropriate therapies was decreased.

The oral administration of NSAIDs was sufficient treatment in 131 cases. In 13 cases opioid-containing analgesics were added, in 22 cases steroids were administered, and muscle relaxants were added in five cases. Thirty-two patients received antibiotics either before confirmation of the diagnosis as LCT or as adjuvant to NSAIDs.

Soft cervical collars were also used in 10 cases. The length of time required for resolution of symptoms was available in 76 cases with an average of 12 days (range ½–42 days). Many authors used non-numerical descriptions like “a few days” or “a few weeks”.

Table 3 summarizes the results of the data of the 242 cases available in the literature, while Table 4 shows the progressive decrease in CRP levels on follow-up of the five cases reported from our institution.

**Table 3.** Summary of the results of the available cases in the literature.

Total number of cases	242
Average age (years)	43 (range 21–81)
Sex (females/males)	129/113
Neck pain (VAS)	100%
Limited ROM*	98.3%
Swallowing complaints	83.72%
Body temperature (available in 36 cases)	37.54 °C (range 36.2–38.9 °C)
Leukocyte count** (available in 48 cases)	11.58 × 10^3^/mm^3^ (range 5.3–21 × 10^3^/mm^3^)
CRP*** (available in 21 cases)	23.66 mg/dL (range 0.3–91.3 mg/dL)
ESR (available in 45 cases)	31.57 mm/h (range 5–98 mm/h)
Calcifications	185 cases (about 76.44%)
Soft tissue swelling/Effusion	190 cases (78.51%)
Duration till subsidence (available in 76 cases)	12 days (range ½–42 days)

VAS = Visual analogue scale for neck pain at presentation,

* ROM = range of motion,

** Leukocyte count (normal range 3.80–9.80 × 10^3^/mm^3^),

*** CRP = C-reactive protein (normal range 0–5 mg/dL).

**Table 4.** The serial resolution of CRP under treatment.

CRP	On admission (mg/L)	2 days after presentation (mg/L)	5 days after presentation (mg/L)	14 days after presentation (mg/L)
Patient 1	7.8	7	5	5.2
Patient 2	38	30	19	7.4
Patient 3	39.7	20.4	15.1	5.1
Patient 4	32.1	51.3	26.1	9.4
Patient 5	4.9		4.7	

## Discussion

LCT is an uncommon condition; however, accurate data regarding its incidence is lacking. Horowitz et al. estimated the mean annual crude incidence of LCT to be 0.50 cases per 100,000 persons-years. Although not common, LCT is probably severely underdiagnosed because LCT symptoms are non-specific, treating physicians are often unfamiliar with it, and it has a self-limiting pathology [8]. The most commonly affected age group is between 30 and 60 years [9, 10].

### Anatomy and pathophysiology

The longus colli muscle lies in the prevertebral space, posterior to the retropharyngeal space. It is the deepest anterior cervical muscle and consists of three parts: upper oblique, vertical, and lower oblique [8]. Upper oblique fibers arise from the anterior tubercles of the transverse processes of the C3–C5 vertebrae and extend to the anterior tubercle of atlas, vertical fibers extend from the bodies of the upper thoracic and lower cervical vertebrae to the bodies of the upper cervical vertebrae, and the lower oblique fibers extend from the front part of the T1–T3 vertebral bodies to the anterior tubercles of the transverse processes of the C5–C6 vertebrae. Its main action is flexion of the neck. Furthermore, the muscle has an important postural function. It counteracts the lordosis increment related to the weight of the head and to the contraction of the dorsal neck muscles. Together with the posterior cervical muscles they form a sleeve that stabilizes the cervical spine in all positions of the head [11–13].

LCT commonly affects the C1/2 level or the upper oblique part of the muscle. Few studies reported a more caudal focus of calcification with the incidence of calcific tendinitis at the origin of the longitudinal part of the muscle mainly at the C4/5 and C5/6 levels [14–16].

The term “retropharyngeal calcific tendinitis” is somewhat misleading as the disease actually begins within the prevertebral space, sometimes but not always extending into the retropharyngeal space. Hence, the term LCT or acute calcific prevertebral tendinitis (ACPT) is preferred [15].

Although the calcifications appear to induce the inflammatory response, the precise mechanism is not yet fully understood. This is similar to the idiopathic nature of calcium hydroxyapatite deposition disease (CHADD), and by definition, LCT falls into the spectrum of CHADD. It has been proposed that calcium deposition may be due to local or systemic metabolic derangements, such as those following ischemia, necrosis, or trauma [15]. That is why a previous history of trauma to the head and neck (e.g. whiplash injury) should be considered [17]. A genetic predisposition has also been hypothesized [16]. The calcifications of LCT also undergo spontaneous resolution. As the calcifications become resorbed, their margins become less well defined, and complete resorption can occur in as little as two weeks. Rupture of these calcific hydroxyapatite crystals provokes an inflammatory response in the surrounding longus colli muscle and this leads to reactive fluid formation in the retropharyngeal space anterior to the muscle [3].

### Clinical presentation

Clinical symptoms of LCT are non-specific and variable including pain and stiffness in the neck (posterior, anteroposterior, and lateral), shoulder and arm pain, odynophagia, dysphagia, a choking sensation, upper back pain, occipital headache, dizziness, nausea, and torticollis. The duration of symptoms before presentation varies between one day and three months [18]. In most cases, neck pain, limitation of cervical spine movement due to pain, and swallowing complaints (odynophagia or dysphagia) comprise the classic triad of symptoms.

The presenting pattern is that of gradually increasing pain in the neck and throat, sometimes with radiation to the occiput. This pain is aggravated by swallowing and motion of the head or neck. There is usually marked limitation of the extension of the neck. There may be mild fever and elevation of the sedimentation rate. The pain usually reaches a maximum at 2–4 days, at which point the patient typically seeks medical help. After reaching its peak, the pain gradually subsides and is usually resolved within 1–2 weeks [19].

The headache associated with LCT generally begins around the same time as the onset of the retropharyngeal tendinitis, significantly worsens with progression of the condition, is aggravated by extension of the neck, rotation of the head and/or swallowing, and is usually associated with tenderness over the spinous processes of the upper three cervical vertebrae [20, 21].

### Imaging investigations

Plain radiographs can reveal an increased soft tissue shadow in front of the upper cervical spine and sometimes amorphous calcifications anterior to the C1/2 vertebrae. CT more clearly demonstrates the calcification and soft tissue swelling. A contrast-enhanced CT scan with tissue phase is the examination of choice for diagnosis confirmation as it reveals best the retropharyngeal edema centered on the calcification and rules out infectious, traumatic, or neoplastic etiologies [20].

A CT-guided biopsy of the prevertebral changes may be recommended in cases where the differentiation from a retropharyngeal abscess is difficult.

MRI can also visualize the edematous longus colli muscle. The diffuse swelling of the muscle is associated with prominent high signal intensities in T2-weighted images [10]. STIR (Short Tau Inversion Recovery) and T1-weighted sequences are preferred to T2-weighted sequences as they make the differentiation of edema or effusion from retropharyngeal infection or spondylitis easier [22]. Also, inflammation of vertebral bone in this disease has been reported. In some cases, there is effusion or synovitis in the atlantoaxial joint, and these can be differentiated with contrast-MRI [23].

Clinically and radiographically, LCT sometimes mimics spondylodiscitis especially when associated with bone marrow edema at the insertion of the muscle [24]. In our case series, we had a case associated with vertebral bone edema C1/2 that mimics a spondylodiscitis or spondylitis.

Other investigations and interventions include CT brain to exclude subarachnoidal hemorrhage or other causes of headache and lumbar puncture to exclude meningitis [17, 25].

### Differential diagnoses

There is a long list of differential diagnoses to consider when a patient presents with the symptoms of LCT. Other conditions to be considered include: retropharyngeal abscess, adenitis, retropharyngeal cellulitis, pharyngitis, acute thyroiditis, traumatic injuries, spondylitis, meningitis, subarachnoid hemorrhage, and rheumatoid conditions.

LCT is also counted among the painful, non-infectious craniofacial and cervical musculoskeletal pathologies, such as temporal tendinitis, Eagle’s syndrome, glossopharyngeal neuralgia, and carotidynia.

Finally, it is also necessary to differentiate radiographically LCT from accessory ossicle, which is a normal variant found in the same location. This ossicle has well-defined borders and no soft tissue swelling. Styloid processes can also be misinterpreted as calcifications [26].

A lack of awareness of this entity and its radiological findings can result in a misdiagnosis and unnecessary interventions, such as the incision and drainage of the retropharyngeal space, especially if retropharyngeal abscess is suspected. This could cause undue discomfort and delay in discharge from the hospital [10].

LCT is a self-limiting condition. Symptoms usually resolve spontaneously within 2–3 weeks. Administration of NSAIDs usually alleviates the acute symptoms. Sometimes a soft cervical collar is prescribed.

## Conclusion

Although LCT is an uncommon condition with non-specific and heterogeneous presentations, spine surgeons with either an orthopedic or a neurosurgical background should be aware of the condition in order to avoid unnecessary investigations or interventions. Of course, priority must first be given to ruling out more serious and possibly life-threatening conditions. Likewise, other spine-related conditions including spondylitis, spondylodiscitis, and traumatic upper cervical injuries must be excluded.

## Conflict of interest

The authors have no conflict of interest.
